# Evaluation and Description of Friction between an Electro-Deposited Coating and a Ceramic Ball under Fretting Condition

**DOI:** 10.3390/ma8084778

**Published:** 2015-07-28

**Authors:** Kyungmok Kim

**Affiliations:** School of Aerospace and Mechanical Engineering, Korea Aerospace University, 76 Hanggongdaehang-ro, Deogyang-gu, Goyang-si, Gyeonggi-do 412-791, Korea; E-Mail: kkim@kau.ac.kr; Tel.: +82-2-300-0288; Fax: +82-2-3158-3189

**Keywords:** fretting wear, electro-deposited coating, friction coefficient, AISI52100

## Abstract

This article describes fretting behavior of zirconia and silicon nitride balls on an electro-deposited coating. Fretting tests are performed using a ball-on-flat configuration. The evolution of the kinetic friction coefficient is determined, along with slip ratio. Experimental results show that the steady-state friction coefficient between ceramic balls (Si_3_N_4_ and ZrO_2_) and an electro-deposited coating is about 0.06, lower than the value between AISI 52100 ball and the coating. After a steady-state sliding, the transition of the friction coefficient is varied with a ball. The friction coefficient for ZrO_2_ balls became a critical value after higher fretting cycles than those for Si_3_N_4_ and AISI 52100 balls. In addition, it is identified that two parameters can describe the transition of the friction coefficient. Finally, the evolution of the friction coefficient is expressed as an exponential or a power-law form.

## 1. Introduction

Frictional contact exists between a ball and a rail comprising an automotive seat slide track. For adjusting a seat, a ball is permitted to roll or slide between two rails. Once the adjustment of a seat is completed, a ball is not allowed to roll or slip. However, auto-engine vibration brings about small amplitude oscillatory movement (called as fretting) between a ball and a rail. The surface of a rail is typically coated by an electro-deposition process for anti-corrosion [1]. Fretting leads to wearing-off of an electro-deposited coating, eventually resulting in decreasing corrosion resistance and increasing squeaking noise.

A conventional ball for automotive seat slide tracks is made of AISI 52100 steel. Ceramic balls are of great interest in the design of seat slide tracks. Particularly, zirconia and silicon nitride can be considered instead of AISI 52100 due to their good tribological properties [2,3]. The kinetic friction coefficient between silicon nitride and steel was found in the range 0.54–0.59 at dry conditions [4]. The friction coefficient between zirconia and steel was measured as about 0.4 with water lubrication [5]. Meanwhile, in the author’s earlier work, the friction coefficient between a ceramic ball and an electro-deposited coating was determined in the range 0.25–0.3 under reciprocal sliding conditions [6]. However, the fretting behavior of ceramic balls was not studied under the conditions similar to those found in automotive seat slide tracks.

In order to describe anti-fretting performance of a coating, appropriate quantities need to be used. Some studies showed that it was possible to describe the friction coefficient evolutions of solid lubricant coatings with parameters in Kachanov-type damage law [7,8,9,10]; it was identified that the change rate of the friction coefficient was described with the damage exponent and the damage rate constant. The influences of surface treatment and coating thickness on the friction coefficient evolution were quantified with the damage rate constant. Meanwhile, the effect of coating material on the friction coefficient evolution was described with the damage exponent. Dissipated energy density was proposed to quantity the amount of fretting damage [11]. It was identified that the energy density can be used for describing fretting durability of a coating. Recently, the ratio of hardness to modulus was used as a tribological parameter to indicate surface resistance to plastic deformation [12,13]. The ratio was found to provide an adequate description of anti-fretting properties of a coating. However, the influence of a counterpart on fretting damage of an electro-deposited coating was not studied with the parameters described above.

In this article, fretting tests were conducted to identify anti-fretting performance of an electro-deposited coating against silicon nitride (Si_3_N_4_) and zirconia (ZrO_2_) balls for use on automotive sliding rails. The coefficient of kinetic friction between an electro-deposited coating and a ceramic ball was determined. The change rate of the friction coefficient was then described as a form of Kachanov-type damage law. Finally, a method was proposed for predicting the friction coefficient evolution of an electro-deposited coating.

## 2. Experimental Details

**Figure 1:**
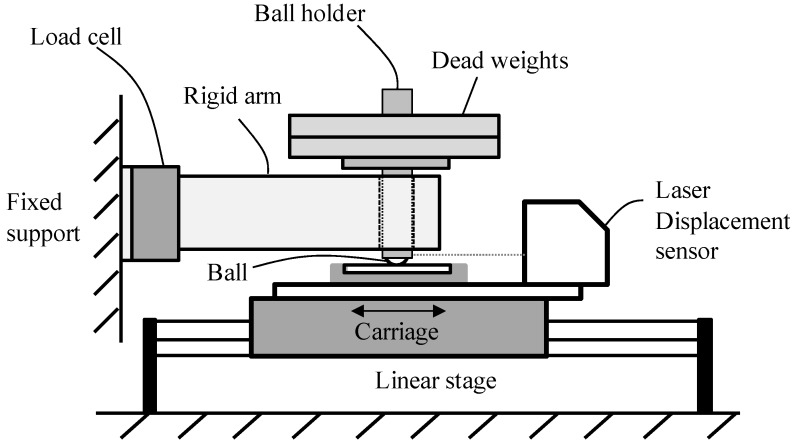
Schematic illustration of the apparatus for fretting testing.

Figure 1 shows the schematic illustration of a fretting testing apparatus. A flat specimen was attached on the carriage of a linear stage. The carriage of a linear stage generated small amplitude oscillatory motion within a pre-described displacement range. A ball was clamped by a ball holder that was permitted to move vertically in a rigid arm. The rigid arm was connected to a load-cell (Interface Inc., moment compensated, Atlanta, GA, USA) attached on the fixed support. A tangential force at the contact between a ball and a flat plate was measured with a load-cell. A normal force was induced to the contact by dead weights. A laser displacement sensor (Keyence Corp., Itasca, IL, USA, LK-081, a resolution of 3 μm and a linearity of ±0.1%) was fixed on the carriage of the linear stage. This sensor measured a relative displacement between a ball holder and a flat specimen on a carriage. The relative displacement was recorded on a program written in *Labview*. A fretting loop was generated and the kinetic friction coefficient was then calculated.

The flat specimen was made of cold-rolled high strength steel: elastic modulus of 205 GPa, *Poisson*’s ratio of 0.28, a tensile strength of 780 MPa, and a yielding strength of 365 MPa. Table 1 shows the chemical composition of the flat specimen.

**Table 1:** Chemical composition (wt %) of the flat specimen provided by the manufacturer.

C	Mn	Si	P	S	Nb	Mo	Cu	Cr	N	Fe
0.07	2.30	1.05	0.02	0.05	0.02	0.02	0.02	0.01	0.03	96.42

An epoxy-based cathodic electro-deposited coating was applied onto the substrate of a specimen with a thickness of 20–30 µm. Condition of electro-deposition was shown in Appendix A. The coating included an epoxy resin with a crosslinker of blocked aromatic isocyanates and with a metal catalyst. Density of a coating layer was 1.3–1.5 g/cm^3^ and pencil hardness was about 5 H (corresponding to Knoop hardness of 34.9). The arithmetic average surface roughness (*R*_a_) of a coated specimen was approximately 0.5 µm.

A ball used for commercial automotive seat slide tracks is AISI 52100 steel with a diameter of 5 mm. In this study, silicon nitride (Si_3_N_4_) and zirconia (ZrO_2_) balls were selected; ball diameter was 5 mm and the arithmetic average surface roughness (*R*_a_) was approximately 0.025 µm. The chemical composition of balls was shown in Appendix B.

In this study, dry fretting tests were conducted at temperature of 24–26 °C and humidity of 60%–70%. Test conditions were a nominal displacement of 0.3 mm, a frequency of 1 Hz, and a normal force of 50 N. The normal force of 50 N was similar to that found on automotive sliding rails. At a normal force of 50 N, the maximum values of *Hertzian* contact pressure were calculated as 2647 MPa for AISI 52100 balls, 2690 MPa for ZrO_2_ balls, and 2998 MPa for Si_3_N_4_ balls (Appendix C).

## 3. Results and Discussion

**Figure 2:**
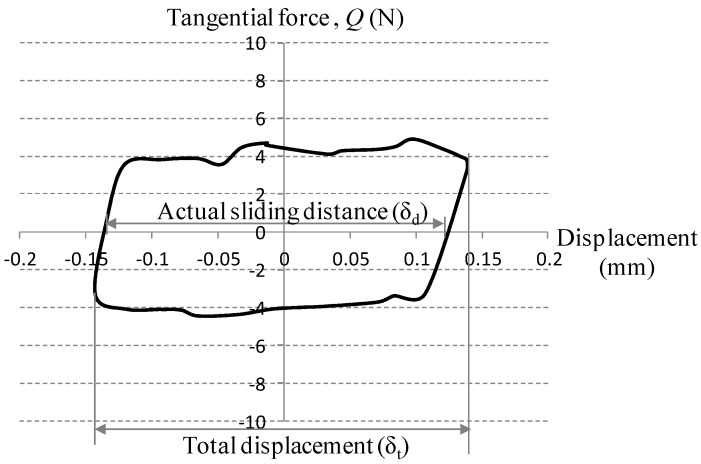
The initial fretting loop for an electro-deposited coating against a conventional ball.

Fretting tests were conducted with Si_3_N_4_, ZrO_2_, and conventional (AISI 52100) balls, respectively. In the course of a fretting test, the kinetic friction coefficient was determined along with a displacement of a moving specimen relative to a ball. The kinetic friction coefficient (μ) was defined as:

(1) $$\text{μ}=\frac{Q_{\max}}{P}$$

where, *Q*_max_ is the maximum tangential force and *P* is imposed normal force in Figure 2. A test was terminated when the friction coefficient reached 0.5.

**Figure 3:**
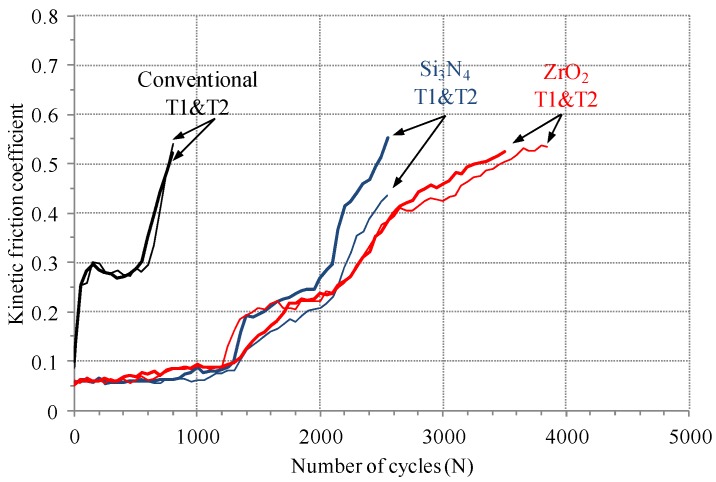
The kinetic friction coefficient evolution between an electro-deposited coating and a ball. “Conventional” denotes AISI 52100 ball. T1 and T2 mean tests 1 and 2, respectively.

**Figure 4:**
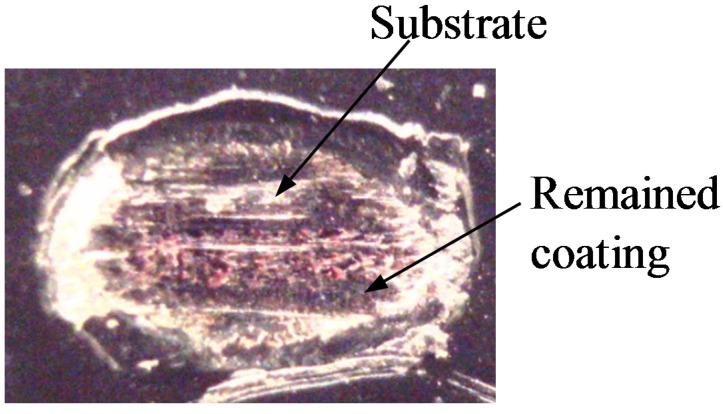
Worn surface image at the friction coefficient of 0.5: An electro-deposited coating against a conventional ball.

Figure 3 shows a friction coefficient evolution between an electro-deposited coating and balls (Si_3_N_4_, ZrO_2_, and AISI 52100) at a normal force of 50 N, a frequency of 1 Hz, and a nominal displacement of 0.3 mm. Good agreement was found between two friction coefficient evolutions with commercial and ZrO_2_ balls. However, there was slight difference between two evolutions with Si_3_N_4_ balls. The variance of the initial coating thickness attributed to such difference between the two evolutions. All initial friction coefficients were lower than 0.1. The friction coefficient for conventional balls (AISI 52100) came to 0.3, and then remained steady up to the 500th cycle. Meanwhile, the initial friction coefficient for Si_3_N_4_, and ZrO_2_ balls were about 0.06. The friction coefficient evolutions for Si_3_N_4_, and ZrO_2_ balls showed a steady-state sliding up to the 1100th cycle. After a steady-state sliding, the friction coefficient for all balls showed strong increase. The friction coefficient for a commercial ball came to 0.5 after 800 cycles, while those for ZrO_2_ and Si_3_N_4_ balls became 0.5 after 3500 and 2500 cycles, respectively. Figure 4 shows the fretted surface image at a friction coefficient of 0.5 for a commercial ball. It was observed that an electro-deposited coating layer was almost removed and the substrate appeared at contact surface.

**Figure 5:**
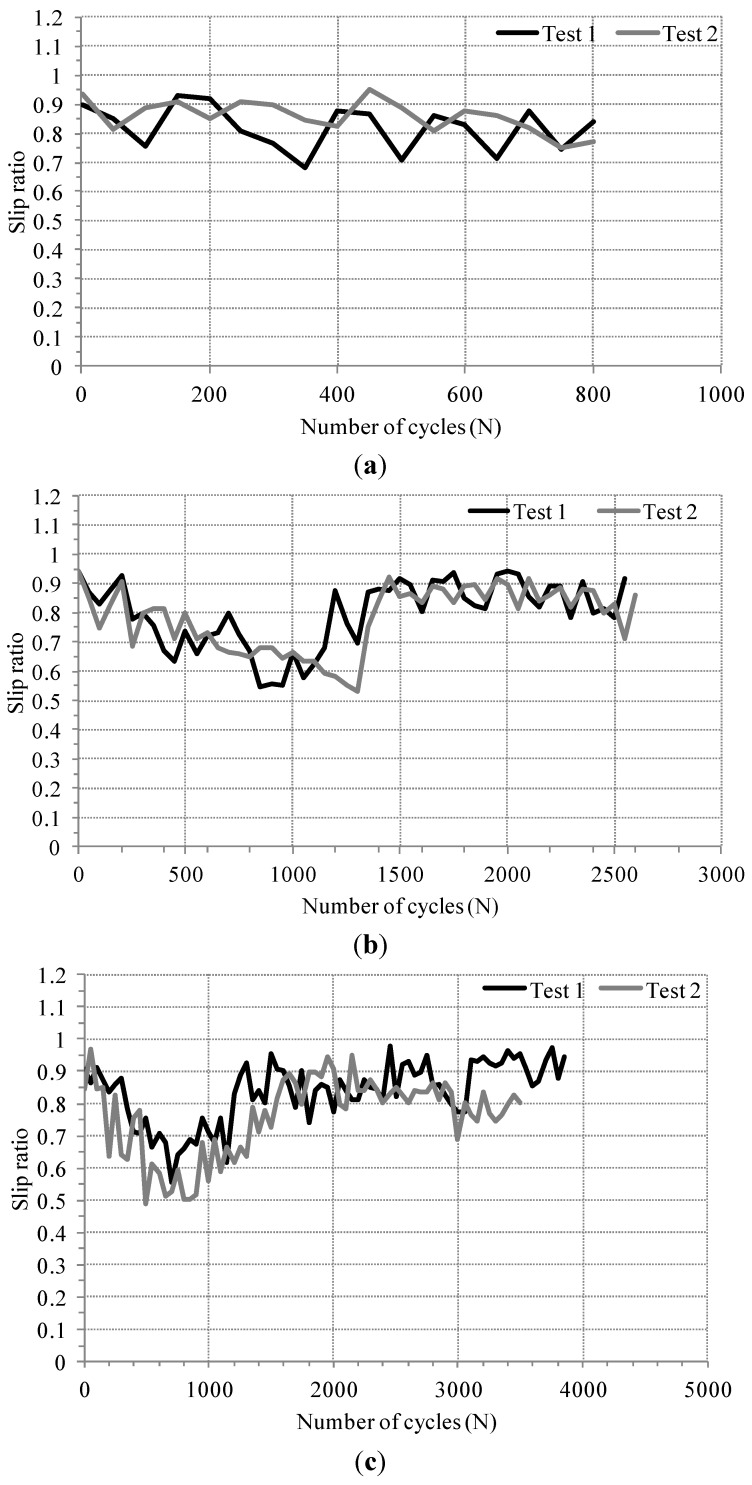
The evolution of slip ratio: (**a**) Conventional (AISI 52100); (**b**) Si3N4; and (**c**) ZrO_2_ balls.

In this study, fretting damage of an electro-deposited coating within a gross slip regime was focused. Slip regimes can be identified with several parameters. In this study, slip ratio was used for identifying a slip regime, proposed in the literature [14]. Slip ratio was defined as an actual sliding distance divided by total displacement in a fretting loop. Figure 5 shows the evolution of slip ratio. All slip ratios for conventional (AISI 52100) and Si_3_N_4_ balls were greater than 0.5 and lower than 0.95, indicating that all fretting tests were completed within a gross slip regime [14]. Although a few slip ratios for test 1 with ZrO_2_ exceeded 0.95, most slip ratio values remained below 0.95.

After a steady-state sliding, the friction coefficient increased with increasing number of cycles, since the substrate appears at contact and surface roughness increases. It was necessary to describe the change of the friction coefficient in order to predict the evolution of the friction coefficient under fretting condition. It is possible to express the friction coefficient change with a help of Kachanov-type damage law [7,8]. That is, the change rate of the friction coefficient (dµ*/*d*N*) can be described with the friction coefficient (µ), as presented in Equation (2).

(2) $$\frac{\text{dμ}}{\text{d}N}=C\times {\text{μ}}^{n}$$

where, *C* is the friction coefficient rate constant and *n* is the friction coefficient exponent.

For the determining the parameters *C* and *n*, the following procedure was employed. First, the measured friction coefficient (µ) was fitted with a power-law function, as presented in Appendix D. The derivative of the friction coefficient, dµ*/*d*N*, was then computed with a fitted curve. Finally, dµ*/*d*N versus* µ was plotted on bilogarithmic scale. On the plot, a straight line presented that the evolution of the friction coefficient obeyed Equation (2). The parameters *C* and *n* in Equation (2) were determined on the evolution curve.

**Figure 6:**
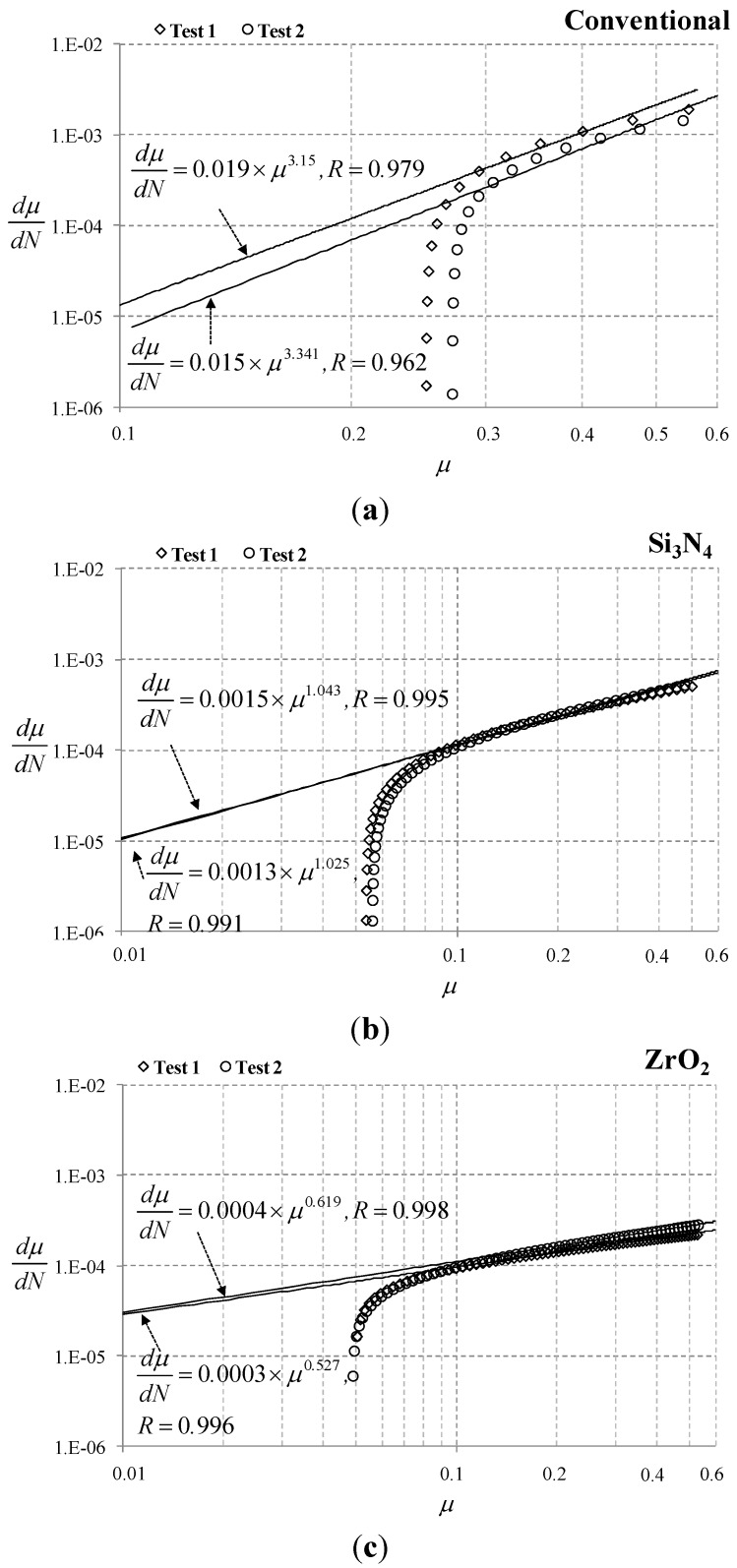
Determination of the parameters *C* and *n* on the log-log scale. “Conventional” denotes AISI 52100. (**a**) Conventional (AISI 52100); (**b**) Si_3_N_4_; and (**c**) ZrO_2_ balls.

Figure 6 shows the plot for determination of the friction coefficient rate constant and the friction coefficient exponent in Equation (2). Reported quality of fit (*R*) showed that Equation (2) provided the adequate description for the growth rate of the friction coefficient. On the log-log plot, the slope of the curve fit was associated with the friction coefficient exponent (parameter *n*), while the Y-intercept was associated with the friction coefficient rate constant (parameter *C*). It was identified that the parameters were varied with respect to a ball. Particularly, the friction coefficient exponent for Si_3_N_4_ ball was close to unity. Meanwhile, the friction coefficient exponents for conventional (AISI 52100) and ZrO_2_ balls were greater than unity and lower than unity, respectively. In the author’s earlier articles [7,9,10], studies were conducted on the correlation between the parameters and experimental conditions under fretting and sliding conditions; the friction coefficient exponent (*n*) was associated with materials of contacting bodies, whereas the friction coefficient rate constant (*C*) was related to surface treatment, normal force and initial coating thickness on coated systems. As shown in Figure 6, it was identified that the friction coefficient exponent was varied with respect to ball material. The friction coefficient rate constant was found to change as well. One of possible reasons might be the variance of initial coating thickness.

If the friction coefficient exponent in Equation (2) is unity, the friction coefficient is expressed as:

(3) $$\text{μ}={\text{μ}}_{1}\times e^{C\cdot (N-N_{1})}$$

where, µ_1_ and *N*_1_ are the average friction coefficient of a steady-state sliding and the final cycle of a steady-state sliding, respectively.

If the exponent is not unity in Equation (2), the relation between the number of cycles (*N*) and the friction coefficient (µ) is given as:

(4) $$\text{μ}={\left(C\cdot (1-n)\left(N-N_{1}\right)+{\text{μ}}_{1}^{1-n}\right)}^{\frac{1}{1-n}}\;n\neq 1$$

The average friction coefficient of a steady-state sliding and the final cycle of a steady-state sliding are presented in Table 2.

**Table 2:** The average friction coefficient of the steady-state sliding and the final cycle of a steady-state sliding. “Conventional” denotes AISI 52100.

Property	Conventional		Si_3_N_4_		ZrO_2_	
	Test 1	Test 2	Test 1	Test 2	Test 1	Test 2
µ_1_	0.28	0.28	0.06	0.06	0.06	0.07
*N*_1_	500	400	750	1000	750	1050

**Figure 7:**
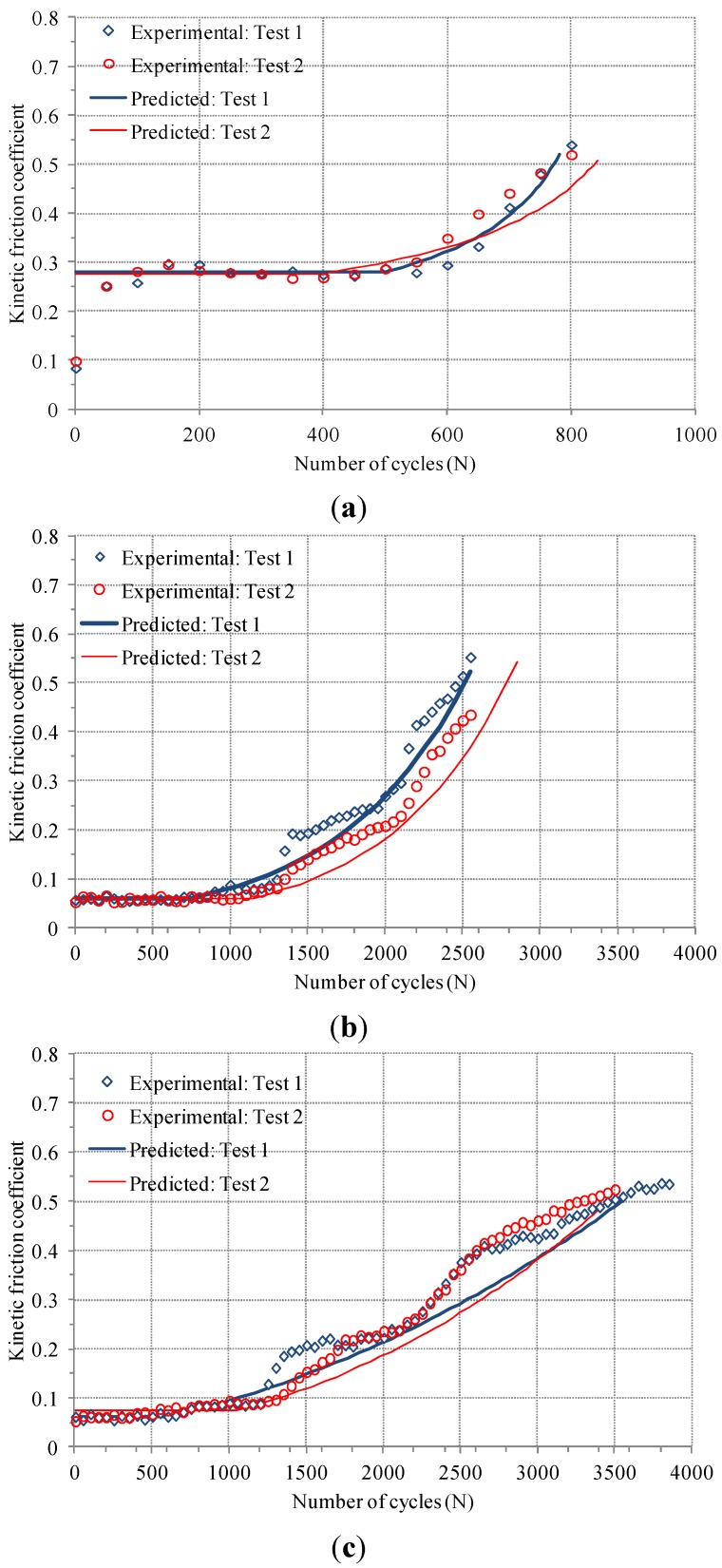
Direct comparison between predicted and measured friction coefficient evolutions: (**a**) Conventional (AISI 52100); (**b**) Si_3_N_4_; and (**c**) ZrO_2_ balls.

Figure 7 shows direct comparison between a predicted friction coefficient evolution and a measured one. Markers were experimental data and smooth curves were predicted friction coefficient evolutions. Good agreement was found between predicted and measured friction coefficient evolutions. It was demonstrated that a proposed method, including Equations (3) and (4), allows predicting a friction coefficient evolution of an electro-deposited coating under fretting condition.

Contact pressure between a ball and a flat specimen has an influence on friction and wear rate of an electro-deposited coating. Apparent relation between the maximum contact pressure and the steady-state friction coefficient was not found in this study due to lack of experimental data. This might be attributed to other factors such as surface roughness, plastic deformation of a coating layer, and the accumulation of wear debris. Thus, further fretting tests need to be conducted at various contact pressures. Wear volume needs to be measured after fretting tests, since the accumulation of wear debris has an influence on friction and dynamic conditions of contact. In addition, other experimental conditions such as temperature and humidity should be taken into account. Above all, remaining work is to find out possible reasons why such a friction coefficient evolution is presented during fretting.

## 4. Conclusions

Fretting tests with zirconia and silicon nitride balls were performed under the condition similar to that found in an automotive seat sliding rails. The following conclusion was then drawn:

- The steady-state friction coefficient of 0.06 was observed between an electro-deposited coating and ceramic (zirconia and silicon nitride) balls in a gross slip regime. They were found to be lower than that of a conventional ball.
- The number of cycles at the friction coefficient of 0.5 for ZrO_2_ balls was 4.38 times as high as that for conventional balls.
- It was identified that the transition of the friction coefficient after a steady-state sliding can be described with two parameters. Friction coefficient evolution of an electro-deposited coating was expressed with the parameters. It was demonstrated that predicted friction coefficients were in good agreement with measured ones.

As further work, measurements of the friction coefficient and wear volume between the coating and other balls (e.g., stainless steel) remains. In addition, experimental factors affecting the parameters need to be studied.

## Appendix

### Appendix A. Condition of Electro-Deposition

**Table A1:** Condition of electro-deposition.

Specification	Value
Solid	15%~22%
Pigment binder ratio	0.15
pH	5.9–6.3
Conductivity	1200–1800 μS/cm
Voltage	230–290 V
ED time	About 180 s

### Appendix B. Chemical Composition (wt. %) of Balls

**Table A2:** Chemical composition (wt. %) of AISI 52100 ball.

Steel	C	Si	Mn	P	S	Cr	Mo
AISI 52100	0.95–1.10	0.15–0.35	0.05	0.025	0.025	1.3–1.6	0.08

**Table A3:** Chemical composition (wt. %) of ceramic balls.

Ceramic	ZrO_2_	Y_2_O_3_	Si_3_N_4_	Y	Al	Ti
ZrO_2_	94.8	5.2	–	–	–	–
Si_3_N_4_	–	–	90.0–92.5	3.0–4.0	3.5–4.5	1.0–1.5

### Appendix C. Determination of Maximum Contact Pressure ($p_{0}$) between a Ball and a Plate with Hertz Contact Theory [15]

$$p_{0}={\left(\frac{6\cdot P\cdot E^{*2}}{{\text{π}}^{3}\cdot R^{2}}\right)}^{\frac{1}{3}}$$

where $\frac{1}{E^{*}}=\frac{1-{\text{ν}}_{1}^{2}}{E_{1}}+\frac{1-{\text{ν}}_{2}^{2}}{E_{2}}$, *P* is normal force, *R* is the radius of a ball, $E_{1}$ and ${\text{ν}}_{1}$ are elastic modulus and *Poisson*’s ratio of a ball, and $E_{2}$ and ${\text{ν}}_{2}$ are elastic modulus and *Poisson*’s ratio of a steel plate, respectively.

### Appendix D. Power-Law Form for Curve Fitting, $\text{μ}={\text{μ}}_{0}+a\times N^{b}$

**Table A4:** Result of curve fitting.

Ball material	Test No.	µ*_0_*	*a*	*b*	*R^2^*
Conventional	1	0.250	2.636 × 10^16^	5.186	0.954
	2	0.270	6.975 × 10^−14^	4.336	0.981
Si_3_N_4_	1	0.053	9.429 × 10^−11^	2.854	0.997
	2	0.056	1.758 × 10^−12^	3.331	0.994
ZrO_2_	1	0.050	9.340 × 10^−7^	1.614	0.989
	2	0.049	8.480 × 10^−8^	1.924	0.992
